# Genome-wide methylation data from *R1* (wild-type) and the transgenic *Dnmt1^Tet/Tet^* mouse embryonic stem cells overexpressing DNA methyltransferase 1 (DNMT1)

**DOI:** 10.1016/j.dib.2020.106242

**Published:** 2020-09-01

**Authors:** Sonal Saxena, Sumana Choudhury, K. Naga Mohan

**Affiliations:** Department of Biological Sciences, BITS Pilani Hyderabad Campus, Jawahar Nagar, Hyderabad 500078, India

**Keywords:** Epigenetics, Schizophrenia, Reduced representation bisulfite sequencing (RRBS), Next generation sequencing (NGS)

## Abstract

Defects in epigenetic mechanisms are well-recognized in multiple neurodevelopmental disorders including Schizophrenia (SZ). In addition to aberrant epigenetic marks, dysregulated epigenetic machinery was also identified among the contributory factors in SZ patients. Among these, overexpression of DNA methyltransferase 1 (DNMT1) was the first to be identified. In this context, *Dnmt1^tet/tet^* (*Tet/Tet*), a mouse embryonic stem cell (ESC) line that overexpresses DNMT1 in ESCs and neurons, was developed to study abnormal neurogenesis. In an attempt to understand whether DNMT1 overexpression is associated with aberrant DNA methylation, we compared the genome-wide methylation levels of *R1* (wild-type) and *Tet/Tet* ESCs and their neuronal derivatives by RRBS. The RRBS data (GSE152817) showed an average mappability of ∼59% and an average coverage of 40X per locus. The data was processed to determine the methylation percentages of target genes and was visualized using the UCSC genome browser. The observed methylation differences were validated by Combined Bisulfite Restriction Analysis (COBRA). The methylome data described here can be used to study the relationship between DNMT1 overexpression, alterations in methylation levels and dysregulation of SZ-associated genes.

## Specifications Table

**Table:** 

Subject	Neurogenetics
Specific Area	Psychiatric epigenetics
Type of data	Tables, Raw sequencing data
How data was acquired	Genomic DNAs were isolated, digested with MspI, gel-purified, treated with bisulfite and subjected to sequencing by RRBS method to obtain 40X coverage of the individual CpG islands
Data format	Raw (files): FASTQ files
	Analyzed (files): bedGraph files
Parameters for data collection	R1 and Dnmt1^tet/tet^ (Tet/Tet) mouse embryonic stem cell lines, Reduced Representation Bisulfite Sequencing, bedGraph files for data visualization.
Description of data collection	Genomic DNAs from the cells above were digested with MspI, ligated to adapters, bisulfite converted and amplified to generate sequencing libraries by RRBS protocol. The libraries were sequenced by Next Generation Sequencing.
Data source location	Birla Institute of Technology and Science, Pilani Hyderabad Campus Hyderabad, India
Data accessibility	Repository name: Gene Expression OmnibusData identification number: GSE152817Direct URL to data: https://www.ncbi.nlm.nih.gov/geo/query/acc.cgi?acc=GSE152817Raw data links for reviewer access: 1. *R1* and *Tet/Tet* ESCs:https://epiquest.s3.amazonaws.com/epiquest_zr2736/VO8JI2S1LTLRLHuw2554zyxzv4W2JN/zr2736_download_fastq.txt 2. *R1* and *Tet/Tet* neurons:https://epiquest.s3.amazonaws.com/epiquest_zr2544/YEZXTG7LQML2WYT6YEWAYRQTXV34AKZ5/09092019_new_rbs/zr2544_download_fastq.txt
Related research article	S. Saxena, P.A. Maroju, S. Choudhury, A. Anne, K.N. Mohan, Analysis of transcript levels of a few schizophrenia candidate genes in neurons from a transgenic mouse embryonic stem cell model overexpressing DNMT1, Gene. 757 (2020) 144934. https://doi.org/10.1016/j.gene.2020.144934.

## Value of the Data

• The dataset described here provides genome-wide DNA methylation patterns at single nucleotide level among CpG islands located at the promoter regions, gene bodies and intergenic regions of *R1* (wild-type) and the transgenic *Dnmt1^tet/tet^* (*Tet/Tet*) ESCs and their neuronal derivatives.
• Since DNMT1 overexpression is one of the etiological factors for schizophrenia (SZ), the RRBS data generated helps in studying the epigenetic basis of DNMT1 overexpression in abnormal neurogenesis.
• The bedGraph files allow visualization of the methylation levels of desired sites in the genome and their comparison across different cell types.

## 1 Data Description

This manuscript describes methylome datasets generated using Reduced Representational Bisulfite Sequencing (RRBS) of genomic DNAs from mouse embryonic stem cells (ESCs) and neurons obtained through their differentiation. The data can be accessed from the Gene Expression Omnibus database with accession number GSE152817. These data were obtained for *R1* (wild-type) ESCs and the transgenic *Dnmt1^tet/tet^* (*Tet/Tet*) ESCs and their respective neuronal derivatives. A total of four RRBS data files, *viz.,* GSM4626846 (*R1* ESCs), GSM4626847 (*Tet/Tet* ESCs), GSM4626848 (*R1* neurons) and GSM4626849 (*Tet/Tet* neurons) were deposited. The data includes the raw reads of all the samples for both paired-end read orientations (read 1 and read 2) in FASTQ format. The sequenced data were deposited at NCBI Sequence Read Archive with accession number SRP267969 under the BioProject accession number PRJNA640537. In order to confirm the integrity of the uploaded datafiles, their md5checksum values were examined (Table 1).

**Table 1:** Details of the files included in the dataset.

Accession Number	Description	File Type	File Names	File Checksum
GSM4626846	*R1* ESCs	FASTQ	zr2736_1_R1.fq	b74d3a9436fa4992572be1b228084e8b
		FASTQ	zr2736_1_R2.fq	7dcc6fe3e612bfed313c1495af9fd30c
		bedGraph	R1 ES RRBS meth calling new.txt	ccf1fee1702c5976894e0c349086f3b4
GSM4626847	*Tet/Tet* ESCs	FASTQ	zr2736_2_R1.fq	0178f0a3d64550dd430b8dfec82912c1
		FASTQ	zr2736_2_R2.fq	e0306f9cbba075f6778b2a0ec94b4781
		bedGraph	Tet ES RRBS meth calling new.txt	1d83c72cc6d335dfe2dac393b3c78e58
GSM4626848	*R1* neurons	FASTQ	zr2544_1_R1.fq	07091d6141e21f079032097199887fa5
		FASTQ	zr2544_1_R2.fq	71aa2e738e249c86639a768bd3ccb468
		bedGraph	R1 neu RRBS meth calling new.txt	7d8e6f71c8c8c2370fbf52946c2e245e
GSM4626849	*Tet/Tet* neurons	FASTQ	zr2544_2_R1.fq	0720d0140ca114e712fdaf55553a2027
		FASTQ	zr2544_2_R2.fq	282d3954c0a397f5ddd16de18d9d7b9f
		bedGraph	Tet neu RRBS meth calling new.txt	e015fcd49cae521b8d5923ea46cfede0

**Fig. 1:**
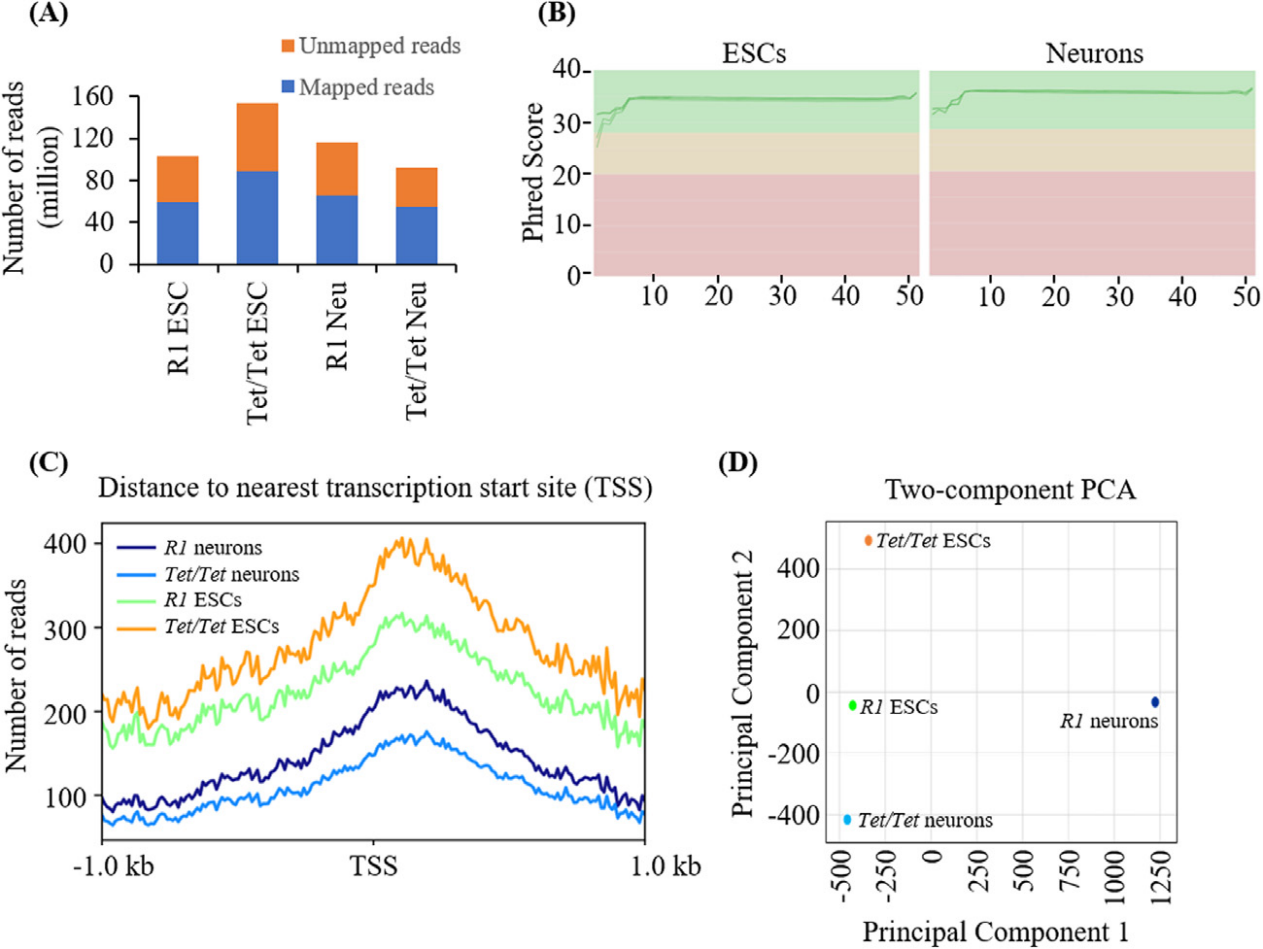
Broad features of RRBS sequencing data from *R1* and *Tet/Tet* ESCs and their corresponding neurons. (A) Number of reads obtained and the proportion of mappable reads in the four cell types. Millions of reads are shown in Y axis against the different cell types on the X-axis. (B) Phred Scores of the RRBS data from the four samples. (C) Distribution of the identified CpG sites with respect to the transcription start sites. (D) Two-component Principal Component Analysis (PCA) of *R1* ESCs, *Tet/Tet* ESCs, *R1* neurons and *Tet/Tet* neurons.

The present dataset was obtained by classic RRBS which covers ≥ 70% of the CpG islands (CGIs) and > 75% of all gene promoters and 1.5 – 2.0 million unique CpG sites that can be identified [1]. A total number of 92.6 – 153.7 million reads were generated from all four samples with 99% conversion rate and 56.8 – 58.7% mappable reads (Fig 1A). Overall, an average coverage of 40X per locus was achieved with Phred Scores > 20 corresponding to > 99% accuracy (Fig 1B). The distribution of RRBS-sequences containing converted and unconverted cytosines for the four cell types suggested that the qualitative distribution of the sequences from 1 kb upstream to 1 kb downstream of the transcription start sites (TSS) is similar (Fig. 1C). Principal component analysis revealed that methylation differences exist between *R1* and *Tet/Tet* ESCs and, between *R1* and *Tet/Tet* neurons (Fig. 1D).

**Fig. 2:**
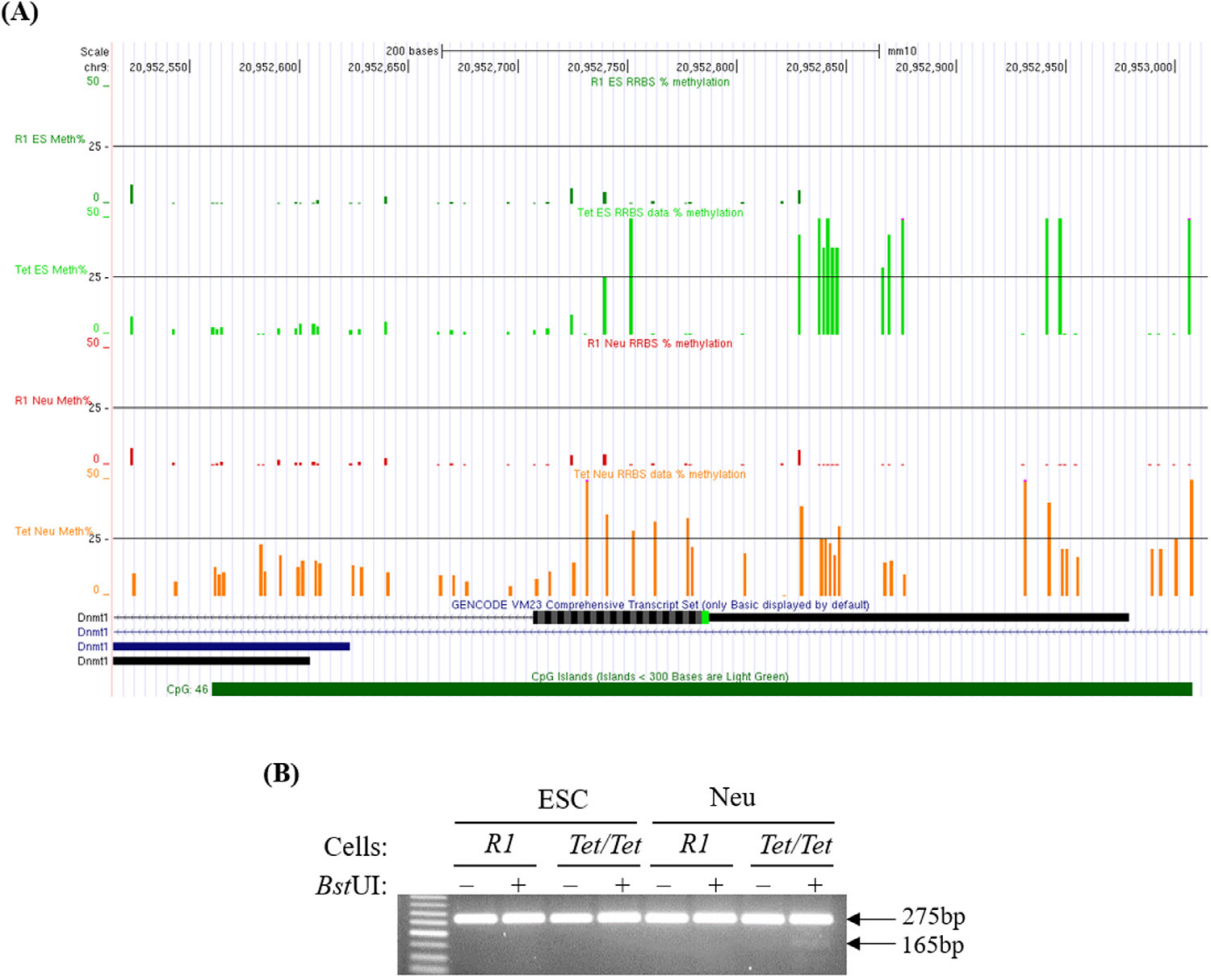
Use of RRBS data in identifying DNA methylation in promoters of genes of interest. (A) DNA methylation data for *Dnmt1* promoter in ESCs and neurons from *R1* and *Tet/Tet* cells viewed in UCSC browser. (B) Validation of DNA methylation data by Combined Bisulfite Restriction Analysis (COBRA). Agarose gel electrophoresis of bisulfite PCR products obtained from biological replicates of ESCs and neurons from *R1* and *Tet/Tet* cells using primers for the indicated 288 bp region. +: reaction mixture was incubated with *Bst*UI enzyme, -: no enzyme control. The presence of a faint 165 bp fragment confirms presence of methylated CpGs in the analyzed genomic region in the DNA from *Tet/Tet* neurons.

Processed data in the form of bedGraph files were provided as supplementary data associated with the accession number GSE152817 (Table 1, GSE152817_RAW.tar). These files were uploaded in the UCSC genome browser for visualization of the RRBS data. As an example, Fig. 2A shows the methylation levels of the CpG sites visualized by querying for the gene *Dnmt1*. Upon focusing on the promoter region, we observed ∼25% increase in methylation of a 288 bp region downstream to the *Dnmt1* promoter in *Tet/Tet* neurons (Fig 2A). Using the same procedure, genome-wide DNA methylation levels can be studied and target genes with dysregulated methylation patterns can be identified.

## 2 Experimental design, materials and methods

The *Dnmt1^tet/tet^* ESC line is a transgenic cell line obtained by knocking in *tet-off* constructs downstream to the *Dnmt1* (DNA methyltransferase 1) promoter of the *R1* ESCs (wild-type) [2]. The cassettes were upstream to the translation start site located in the exon1 of *Dnmt1*. As a result, the *Tet/Tet* ESCs overexpressed DNMT1 and the resultant neurons also showed increased levels of the enzyme [3]. The main purpose of the study was to generate nucleotide level information on the cytosine methylation levels of the genomes of *R1* ESCs, *Tet/Tet* ESCs, *R1* neurons and *Tet/Tet* neurons. DNAs from all the four cell types were isolated by standard procedures and subjected to RRBS analysis (Section 2.2). The RRBS data described here enables analysis of DNA methylation levels of target sequences in *R1* and *Tet/Tet* ESCs and neurons. In future, the data enables identification of target DNA sequences subject to differential methylation due to increased DNMT1 levels. Such information is important to study the role of DNMT1 overexpression in abnormal neurogenesis.

### 2.1 Cell culture

All reagents were of tissue culture or molecular biology grade and obtained from vendors such as ThermoFisher (USA), Merck (Germany), Takara Biotech (USA), HiMedia (India) and New England BioLabs (USA). ESCs were grown in ES media (Knockout DMEM/F-12 with 10% ESC-grade FBS, 1X Non-Essential Amino acids, 1X Glutamax, 1X Penicillin/Streptomycin, 100 µM β-meracptoethanol, 10 ng/ml Leukemia Inhibitory factor) till confluence in 0.1% Gelatin coated cell culture dishes. In case of *Tet/Tet* ESCs, the medium was supplemented with G418 (150 µg/ml) and puromycin (1 µg/ml). After reaching confluence, the cells were treated with 1X Trypsin-EDTA for ten minutes to obtain single cell suspensions. The cells were plated onto non-adherent dishes containing embryoid body medium (ES medium without Leukemia Inhibitory Factor) to obtain EBs after two days. The EBs were differentiated using NDiff 227 medium on cell culture-treated dishes for 10–12 days. At the end of 12 days, a majority of the cells differentiated into neurons with well-branched neurites. A more detailed description of the differentiation process is given in Saxena et al. [3,4].

### 2.2 Extraction of DNA and reduced representational bisulfite sequencing (RRBS)

The RRBS was performed at Zymo Research (USA) using genomic DNAs from *R1* and *Tet/Tet* ESCs and their neuronal derivatives. Genomic DNAs were isolated using SDS-proteinase K digestion and phenol: chloroform extraction method and quantified using Nanodrop® [5]. The method followed for generating RRBS data was adapted from Meissner et al. [1]. About 200 ng each of the DNA samples were digested with 30 units *Msp*I and purified using DNA Clean and Concentrator™-5 (Zymo Research). The fragments were ligated to pre-annealed adapters containing 5′-methylcytosine instead of cytosine as per Illumina's specified guidelines. Adapter -ligated fragments were purified using DNA Clean and Concentrator™-5 and then treated with bisulfite using EZ DNA methylation Lightening Kit (Zymo Research). The converted DNAs were amplified with adapter-specific primers. The amplified products were analyzed by bioanalyzer to ensure correct size distribution and the PCR products were sequenced using next generation sequencing technology using Illumina HighSeq 1500 platform.

Sequence reads were identified using Illumina base calling software and the sequences corresponding to the adapters were removed using TrimGalore 0.6.4 software [6]. The effect of trimming and overall quality distributions of the data were assessed by FASTQC 0.11.8 software [7]. The sequences were mapped to mm10 mouse genome using Bismark 0.19.0 software [8]. Methylated and unmethylated read totals for each CpG site were called using MethylDackel 0.3.0 software [9]. Methylation level of each sampled cytosine was estimated as the number of reads reporting a cytosine divided by the total number of reads reporting cytosine or thymine. In order to identify significant differentially methylated cytosines, Fisher's exact test was performed on sequences with a minimum read coverage of five in each sample with at least 10% difference in the level of methylation. Genes with significant differences in the level of methylation with a *p*-value < 0.05 were identified by summing up all the CpG sites in one gene and the value was used to determine if there is hypomethylation or hypermethylation.

### 2.3 Visualization of methylation data in UCSC browser

The sequence data was converted to bedGraph files and were uploaded as custom tracks in the UCSC genome browser [10]. The methylation levels were displayed in the UCSC browser as vertical bars. For display of methylation levels, the Y-axis in the UCSC settings can be set from 0 to 100 or a desired range, with 100 representing complete methylation (100%) and zero representing no methylation. In order to distinguish the individual tracks, the track files were coded in different colours. The level of methylation for the individual CpG sites for a given genomic region were visualized in the UCSC genome browser by entering the chromosome coordinates or gene name in the query box.

## Validation of methylation differences by Combined Bisulfite Restriction Analysis (COBRA)

The RRBS data generated here and the procedure in general provides an average of 10X - 40X coverage of the target sequences. However, an independent validation of differences in methylation identified by RRBS data is needed as confirmatory evidence. Combined Bisulfite Restriction Analysis (COBRA) is a method that provides an overall estimate of the level of methylation using a larger number of molecules than sampled by RRBS or sequencing of cloned PCR products obtained from bisulfite-treated genomic DNA [11]. In COBRA, specific primers for target sequences under investigation are used to amplify bisulfite-treated DNA. The obtained PCR products are gel-purified for incubation with restriction enzymes containing one or more CpGs in their recognition sites. Unmethylated sequences are converted by bisulfite into uracils and are observed as thymines in the amplified products. Methylated sequences on the other hand are resistant to the conversion and are retained as cytosines and CpGs in the amplified products. Therefore, the extent of digestion of the PCR products by the restriction enzymes mentioned above allows an approximation of methylation levels from a large number of target sequences in the sample. Fig. 2B shows validation of the methylation difference identified in the 500 bp region downstream to the *Dnmt1* promoter in *Tet/Tet* neurons. Agarose gel electrophoresis of samples incubated with the enzyme *Bst*UI (CGCG) suggested digestion of a small proportion of the PCR products obtained from bisulfite treated DNA from *Tet/Tet* neurons but not from the other three cell types. Therefore, the COBRA results confirmed the DNA methylation difference downstream to *Dnmt1* promoter region identified by RRBS (Fig. 2A).

## Declaration of Competing Interest

The authors declare no competing financial interests or personal relationships that could have appeared to influence the work reported in this paper.
